# Angiotensin-Converting Enzyme Insertion/Deletion Polymorphism and Susceptibility to Osteoarthritis of the Knee: A Case-Control Study and Meta-Analysis

**DOI:** 10.1371/journal.pone.0161754

**Published:** 2016-09-22

**Authors:** Chin Lin, Hsiang-Cheng Chen, Wen-Hui Fang, Chih-Chien Wang, Yi-Jen Peng, Herng-Sheng Lee, Hung Chang, Chi-Ming Chu, Guo-Shu Huang, Wei-Teing Chen, Yu-Jui Tsai, Hong-Ling Lin, Fu-Huang Lin, Sui-Lung Su

**Affiliations:** 1 School of Public Health, National Defense Medical Center, Taipei, Taiwan, ROC; 2 Division of Rheumatology/Immunology/Allergy, Department of Internal Medicine, Tri-Service General Hospital, National Defense Medical Center, Taipei, Taiwan, ROC; 3 Department of Family and Community Medicine, Tri-Service General Hospital, National Defense Medical Center, Taipei, Taiwan, ROC; 4 Department of Orthopedics, Tri-Service General Hospital, National Defense Medical Center, Taipei, Taiwan, ROC; 5 Department of Pathology, Tri-Service General Hospital, National Defense Medical Center, Taipei, Taiwan, ROC; 6 Department of Pathology and Laboratory Medicine, Kaohsiung Veterans General Hospital, Kaohsiung, Taiwan, ROC; 7 Department of Physiology and Biophysics, National Defense Medical Center, Taipei, Taiwan‏, ROC; 8 Division of Thoracic Surgery, Tri-Service General Hospital, National Defense Medical Center, Taipei, Taiwan, ROC; 9 Department of Radiology, Tri-Service General Hospital, National Defense Medical Center, Taipei, Taiwan, ROC; 10 Department of Medicine, Cheng Hsin General Hospital, Taipei, Taiwan, ROC; 11 Department of Medicine, Tri-Service General Hospital, National Defense Medical Center, Taipei, Taiwan, ROC; Victoria University, AUSTRALIA

## Abstract

**Background:**

Studies of angiotensin-converting enzyme insertion/deletion (ACE I/D) polymorphisms and the risks of knee osteoarthritis (OA) have yielded conflicting results.

**Objective:**

To determine the association between ACE I/D and knee OA, we conducted a combined case-control study and meta-analysis.

**Methods:**

For the case-control study, 447 knee OA cases and 423 healthy controls were recruited between March 2010 and July 2011. Knee OA cases were defined using the Kellgren-Lawrence grading system, and the ACE I/D genotype was determined using a standard polymerase chain reaction. The association between ACE I/D and knee OA was detected using allele, genotype, dominant, and recessive models. For the meta-analysis, PubMed and Embase databases were systematically searched for prospective observational studies published up until August 2015. Studies of ACE I/D and knee OA with sufficient data were selected. Pooled results were expressed as odds ratios (ORs) with corresponding 95% confidence intervals (CI) for the D versus I allele with regard to knee OA risk.

**Results:**

We found no significant association between the D allele and knee OA [OR: 1.09 (95% CI: 0.76–1.89)] in the present case-control study, and the results of other genetic models were also nonsignificant. Five current studies were included, and there were a total of six study populations after including our case-control study (1165 cases and 1029 controls). In the meta-analysis, the allele model also yielded nonsignificant results [OR: 1.37 (95% CI: 0.95–1.99)] and a high heterogeneity (I^2^: 87.2%).

**Conclusions:**

The association between ACE I/D and knee OA tended to yield negative results. High heterogeneity suggests a complex, multifactorial mechanism, and an epistasis analysis of ACE I/D and knee OA should therefore be conducted.

## 1. Introduction

Knee osteoarthritis (OA) is characterized by a highly catabolic state, chondrocyte apoptosis, articular cartilage degeneration, morphologic changes to the subchondral bone, and damage to the surrounding synovium [1–5]. Multiple factors, such as ageing, genetic, hormonal, and mechanical factors, contribute to OA onset and progression [5–9]. Prior research suggests that OA is primarily influenced by genetic risk factors due to common population polymorphisms in multiple genes [10–16]. This heritability of OA development was once estimated to be as high as 65% [17,18].

Bradykinin is an inflammatory nonapeptide vasodilator and has a role in pain and inflammation mainly mediated via its receptor [19]. Previous evidence suggested an important role of bradykinin in the generation of pain, swelling, and cellular damage associated with inflammatory joint disease, including OA [20,21]. The possible mechanism may be via a decrease in subchondral bone remodeling and an increase in cartilage thickness. Moreover, it can increase levels of cartilage proteoglycans and type II collagen [21]. Bellucci et al. (2009, 2013) further proposed a correlation between the presence of bradykinin in the synovial fluid of OA knees and cartilage degradation and a participatory role of bradykinin in OA pathology [19,22]. Finally, the epidemiological evidence demonstrated a positive correlation between bradykinin and the synovitis score and a higher detection rate of bradykinin among several pain-related mediators [23].

Angiotensin-converting enzyme (ACE, EC3.4.15.1) is a membrane metallopeptidase that converts angiotensin I to the potent vasoconstrictor, angiotensin II [24,25]. ACE plays a role in the cross talk between the renin–angiotensin system (RAS) and the kallikrein–kinin system. ACE not only converts angiotensin I to angiotensin II but also metabolizes bradykinin, which is a strong vasodilator, to form inactive bradykinin 1–5. This phenomenon has been demonstrated through *in vivo* experimental studies, which demonstrated that ACE inhibitor treatment decreased the blood angiotensin II concentration but increased the blood bradykinin concentration in normal human subjects and dogs [26–29].

Given the key role of ACE in the RAS, sufficient evidence has led to suspicion of a relationship between ACE polymorphisms and OA. One of the most important ACE polymorphisms is a 287-bp insertion/deletion in intron 16 (ACE I/D), with this angiotensin-ACE I/D genotype associated with plasma, cellular, and tissue ACE levels. Plasma ACE levels are highest in subjects with the DD genotype, followed by subjects with the ID genotype and lowest in subjects with the II genotype [30–33]. According to the above mechanism, we supposed the D allele was thought to be a protective factor against knee OA, because D allele carriers have higher ACE levels and therefore lower bradykinin concentrations, whereas the I allele is considered a risk factor.

Although many genome wide association studies investigated SNP–disease association in OA [34,35], they were unable to find any evidence of ACE I/D because this locus is a structural variant. Moreover, no meta-analyses have been carried out on this subject. Only a limited number of studies have investigated the link between OA and the ACE I/D polymorphism [36–40]. In reports by Bayram et al. (2011) and Inanir et al. (2013), the DD genotype of the ACE gene I/D polymorphism was associated with knee OA in a Turkish study population [38,39]. Poornima et al. (2015) observed the same phenomenon in an Asian Indian population [40]. In contrast with our hypothesis, the above three studies considered that D allele carriers had the greater risk of OA. However, although Hong et al. (2003) and Shehab et al. (2008) found a negative association between ACE alleles and knee OA, carriers of the I allele had the greater risk of OA [36,37]. Given these inconclusive findings in the literature, we attributed the high heterogeneity to small sample sizes (sample sizes of those studies ranged from 200 to 421 [36–40]). To resolve this dispute with regard to consistent evidence, we proposed a case–control study with a sample size of >800 subjects. Moreover, our study was combined with a concurrent meta-analysis of the present and previous studies to investigate whether the I allele at intron 16 in ACE contributes to knee OA susceptibility.

## 2. Materials and Methods

### 2.1 Case–control study

#### 2.1.1 Sample size and ethical issues

Before starting the study, an appropriate sample size was estimated using formulas developed by Fleiss et al. [41]. The settings used were as follows: a two-sided test with a power (1 − β) of 0.8 at a significance level of 0.05, and a ratio of controls to cases equal to 1; the hypothetical proportion of controls with exposure was 30 [42] with at least an odds ratio (OR) to be detected of 1.5 [43]. Based on these settings, the minimum study sample size required was 850 subjects.

Using this number, we initiated a population-based study at the Tri-Service General Hospital (TSGH), a medical teaching hospital of the National Defence Medical Centre in Taipei, Taiwan. The project was reviewed and approved by the institutional ethical committee of the Tri-Service General Hospital (TSGH-100-05-023). Individuals willing to participate in this study after receiving a full explanation from investigators were enrolled, and all participants included in this project provided signed, informed consent. This project was described in our previous report [44].

#### 2.1.2 Subjects

We recruited potential participants at the Health Management Centre of TSGH from those participating in a check-up program. The Taipei city senior medical check-up program is a governmental welfare program provided for people aged 65 years or older and who have been registered residents in Taipei city for more than 1 year. Accordingly, we began to release related recruitment information in March 2010. Participants who met the following criteria were excluded from this study: (1) patients who had undergone knee surgery (e.g., total knee arthroplasty) and (2) those unable to provide a sufficient blood sample. Demographic data included age, gender, and body mass index (BMI: kg/m^2^) and were collected from medical records. A total of 870 independent subjects (400 men and 470 women) aged 65 years or older [mean (standard deviation; SD) age = 74.1 (6.9) years] participated in this study up to July 2011.

#### 2.1.3 Radiographic assessment

All participants underwent a radiographic examination of both knees with anterior–posterior and lateral views analyzed as well as weight bearing and foot-map positioning recorded. Knee radiographs were read and scored by two readers, a radiologist and rheumatologist blinded to the patients’ clinical information, using the Kellgren–Lawrence (KL) grading system [45]. In the KL system, radiographs receive scores of 0–4 points. If the readers assigned different KL grades, we recruited a third interpreter to confirm the final grade. For patients with different KL grades in each knee, the more advanced grade was used for evaluation. We used a radiographic KL grade of ≥2 to define knee OA. According to the above classification, the study included 447 knee OA patients and 423 healthy controls.

#### 2.1.4 Genomic DNA extraction and genotyping

Genomic DNA was extracted from peripheral blood samples using standard procedures for proteinase K (Invitrogen, Carlsbad, CA, USA) digestion and phenol/chloroform extraction. ACE I/D polymorphisms were screened using the polymerase chain reaction (PCR) according to a previously described protocol [46]. The primers were also described previously [47]. To exclude this possibility, all DD homozygotes were retyped using an I-specific sense primer as previously described [48]. The PCR program included the following steps: initial denaturation at 95°C for 5 min; 35 cycles of denaturation at 95°C for 30 s, annealing at 58°C for 30 s, and extension at 72°C for 30 s and a final extension at 72°C for 10 min. Genotyping was performed under blinding of the case or control status. Two independent investigators interpreted images of each gel, and all ambiguous samples were analyzed twice. To validate the genotyping results, at least 10% of samples were randomly selected for repeated genotyping.

#### 2.1.5 Statistical analysis

Continuous demographic variables were evaluated using a Student’s t test and reported as means ± SDs. The Hardy–Weinberg equilibrium (HWE) was assessed using a goodness-of-fit χ^2^ test performed to identify possible genotyping errors among the controls of each study. Genotypes and allelic frequencies were compared between patients with knee OA and healthy controls using the χ^2^ test or Fisher’s exact test where appropriate. Logistic regression was used to estimate ORs and 95% confidence intervals (CIs) as a measure of the association with the risk of knee OA. Allele type, genotype, and dominant/recessive models were used to calculate the association between genetic polymorphism and knee OA risk. To avoid the multiple comparison problem, we tested the global p value via two robust tests, MAX3 [49,50] and the genetic model selection (GMS) [51]. This study considered a p value of <0.05 as significant for all analyses. Statistical analyses were carried out using R software, version 3.2.3 (R Project for Statistical Computing, Vienna, Austria) with the “Rassoc” package [52].

### 2.2 Meta-analysis

#### 2.2.1 Search methods and criteria for study consideration

The PRISMA checklist and Meta-analysis on Genetic Association Studies Checklist is described in S1 and S2 Tables [53]. This study focused on a general population and aimed to compare OA risks between individuals carrying the major (I) and minor (D) alleles of ACE I/D. To identify relevant studies, English-language articles in PubMed and Embase were searched using relevant text words and medical subject headings that included all spellings of ACE I/D and OA (detailed search strategy and records are shown in S3 Table). All articles published from the dates of inception of these medical databases to January 2016 were included.

All related studies that assessed the association between ACE I/D polymorphisms and the risk of knee OA were considered for inclusion. The criteria for study inclusion were as follows: (1) cross-sectional surveys or case–control studies, (2) OA defined as a KL grade of ≥2, and (3) a detailed distribution of ACE genotypes. If the published data was incomplete, we made attempts to contact the authors for further information.

#### 2.2.2 Data extraction and quality assessment

Two reviewers (Chin Lin and Wen-Hui Fang) independently extracted data and assessed the risk of bias. For each article, we recorded the first author’s name, year of publication, ethnicity of the study population, definition of the case group, and population characteristics (mean age, proportion of male subjects, BMI, and ACE I/D genotype distribution). All extracted papers were assessed using the Newcastle–Ottawa Scale (NOS) [54], and all received scores >5 points.

#### 2.2.3 Statistical analysis

The population characteristics of each included study are presented as means or proportions where appropriate. Our meta-analysis examined the association between ACE I/D polymorphisms and the risk of knee OA in each study using ORs with 95% CIs. The τ^2^ statistic, which was estimated using the DerSimonian–Laird method, was used to assess heterogeneity, and a random-effects model was used to calculate the weighed effect size. Three common genetic models, including allele type, dominant, and recessive models, were used to calculate the association between genetic polymorphism and the risk of knee OA. Moreover, we performed a genotype model with three comparisons (ID vs. II, DD vs. II, and DD vs. ID) for comprehensively examining the association between ACE I/D and knee OA. Because the genotype model tested three times in an association, the Bonferroni method was used to correct these for significant values (divided by 3).

Egger’s regression and a funnel plot were used to test the symmetry of pooled results [55]. I^2^ was calculated with the Cochrane Q test and used to quantify heterogeneity; an I^2^ value >50% indicated a moderate to high heterogeneity [56]. Moreover, meta-analyses commonly remove studies with HWE violations [57,58]; we therefore performed a sensitivity analysis according to this rule.

Meta-regression using an average summary value is used to explore the source of heterogeneity. According to our previous studies, the average summary value of the case group can be used to build a model and can help to estimate the interaction effect [59,60]. An interaction effect is determined using the OR and defined as the ratio between ORs per 1 unit. For example, if the OR of the association between ACE I/D and knee OA risk is 3 in the Arab subgroup and 2 in the Asian subgroup, the moderate effect of ethnicity would be 3/2 or 1.5. Possible moderators (ethnicity, age, gender, and BMI) were tested to explore heterogeneity.

This study considered a p value of <0.05 to be significant for all analyses. Statistical analyses were conducted using the “metafor” [61] and “meta” [62] packages of R software, version 3.2.3.

## 3. Results

### 3.1 Case–control study

There were 870 samples in this study, and 83 (9.5%) ambiguous calls were identified and re-genotyped. After the first round of genotyping, we randomly selected 96 (11.0%) repeated samples for re-genotyping. The reproducibility rate of analyzing ACE I/D in this study was 100%.

The characteristics of subjects according to the severity of knee OA are shown in Table 1. We included 447 cases with a mean age of 74.9 ± 7.1 years (253 men and 194 women) and 423 controls with a mean age of 73.3 ± 6.6 years (217 men and 206 women). The mean age was significantly higher among the cases relative to the controls (p = 0.001), whereas the gender distributions did not differ significantly (p = 0.117). Approximately 80% of cases had moderate knee OA (KL grade = 2), whereas approximately 20% had serious knee OA (KL grade > 2). Moreover, the case group had a significantly higher BMI relative to the control group (p = 0.005).

**Table 1:** Characteristics of subjects with knee osteoarthritis and control subjects.

		Case (N = 447)	Control (N = 423)	p value
**Sex**	Female	253(56.6%)	217(51.3%)	0.117
	Male	194(43.4%)	206(48.7%)	
**Age (years)**		74.9±7.1	73.3±6.6	0.001
**Height (cm)**		158.4±8.1	158.3±11.4	0.881
**Weight (kg)**		61.6±10.2	60.5±10.4	0.116
**BMI (kg/m**^**2**^**)**		24.5±3.3	23.9±3.0	0.005
**K–L**	0		182(43%)	
	1		241(57%)	
	2	357(79.9%)		
	3	87(19.4%)		
	4	3(0.7%)		

BMI: body mass index; K–L: Radiographic assessment result by Kellgren–Lawrence grading system.

We further used the global test and four types of genetic models to test the association between ACE I/D and knee OA; the results are shown in Table 2. The D allele frequencies were 35.9% and 33.9% among cases and controls, respectively. The global test of the association of ACE I/D and knee OA was not significant [p value = 0.3167 (MAX3) and 0.4370 (GMS)], and the association between the D allele and knee OA was also nonsignificant [OR: 1.09 (95% CI: 0.76–1.89)]. Moreover, we also evaluated the results from the codominant, dominant, and recessive models and only obtained nonsignificant results. Therefore, we found no association between ACE I/D and knee OA in our case–control study. We also stratified patients by the severity of osteoarthritis and found no significant findings in any of the models (details were shown in S4 Table). To further enhance the level of evidence, we performed a meta-analysis that included our case–control study.

**Table 2:** Angiotensin-converting enzyme insertion/deletion (I/D) genotype frequencies in cases and controls.

	Case	Control	Odds Ratio (95% CI)	p value
**Allele**				
I Allele	573(64.1%)	559(66.1%)	1	0.283
D Allele	321(35.9%)	287(33.9%)	1.09(0.76–1.89)	
**Genotype**				0.298
II	175(39.2%)	185(43.7%)	1	
ID	223(49.9%)	189(44.7%)	1.25(0.94–1.66)	
DD	49(10.9%)	49(11.6%)	1.06(0.68–1.65)	
**Dominant**				
II	175(39.2%)	185(43.7%)	1	0.880
DD + ID	272(60.8%)	238(56.3%)	1.21(0.92–1.58)	
**Recessive**				
II + ID	398(89.1%)	374(88.4%)	1	0.118
DD	49(10.9%)	49(11.6%)	0.94(0.62–1.43)	

CI: confidence interval

The p value of global analysis: 0.3167 (MAX3) and 0.4370 (GMS)

### 3.2 Meta-analysis

**Fig 1:**
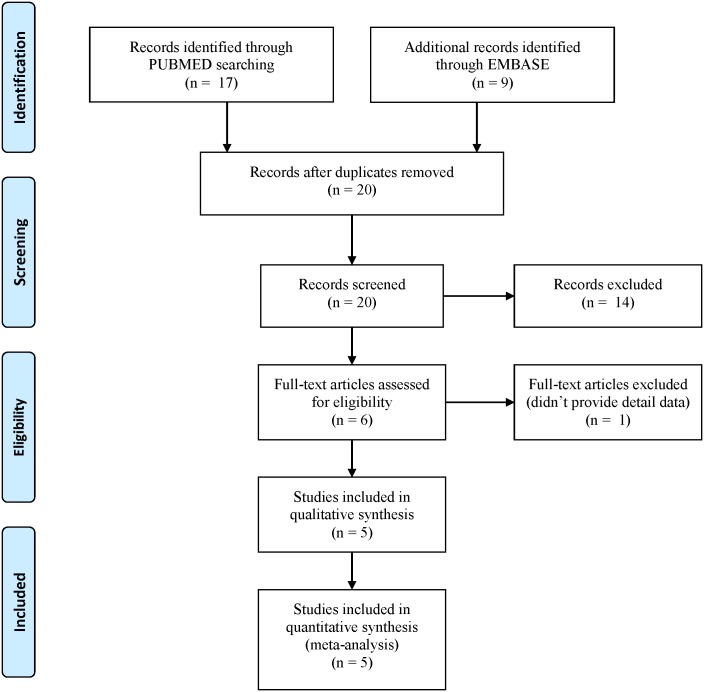
Flow diagram of the identification process for eligible studies.

Fig 1 presents the overall study identification process. Our search strategy returned 17 and 9 records from PubMed and Embase, respectively. Twenty records remained after removing duplicates during the title and abstract review, and 14 of the 20 records were found to be unrelated to the topic (details were shown in S3 Table). Of the remaining six papers, one paper did not have sufficiently detailed data for the analysis. Accordingly, five studies were included in our meta-analysis [36–40], and detailed data are presented in Table 3. Our meta-analysis therefore comprised three Asian studies and three Arab studies, including our case–control study. It is worth mentioning that our study population had the highest mean age, highest proportion of men, and lowest mean BMI. We subjected each control group to HWE testing. Interestingly, none of the Arab studies passed this test. Therefore, the results from the Asian subgroup will represent the results of our sensitivity analysis. Accordingly, we will not separately present the results of the sensitivity analysis.

**Table 3:** Summary of studies included in the meta-analysis.

Study	Country	Ethnicity	HWE test	Case						Control					
				Male (%)	Mean age	Mean BMI	DD	ID	II	Male (%)	Mean age	Mean BMI	DD	ID	II
This study	Taiwan	East Asian	0.998	43.4	74.9	24.5	49	223	175	48.7	73.3	23.9	49	189	185
Poornima et al., 2014	India	South Asian	0.799	32.0	42.4	31.4	44	38	18	31.0	42.2	25.9	22	46	32
Inanir et al., 2013	Turkey	Arabian	0.013	27.1	58.0		77	107	37	32.5	53.0		45	77	78
Bayram et al., 2010	Turkey	Arabian	0.045	27.1	54.2	28.0	81	51	8	28.3	44.6	25.3	24	20	16
Shehab et al., 2008	Kuwait	Arabian	<0.001	11.3	57.1	31.7	70	22	23				74	18	19
Hong et al., 2003	Korea	East Asian	0.292	33.8	58.6	25.2	23	68	51				33	58	44

HWE test: Hardy–Weinberg equilibrium test by a chi-square test with 2 degrees of freedom; BMI: body mass index; DD: the number of subjects carrying the DD genotype in ACE I/D; ID: the number subjects carrying the ID genotype in ACE I/D; II: the number subjects carrying the II genotype in ACE I/D.

**Fig 2:**
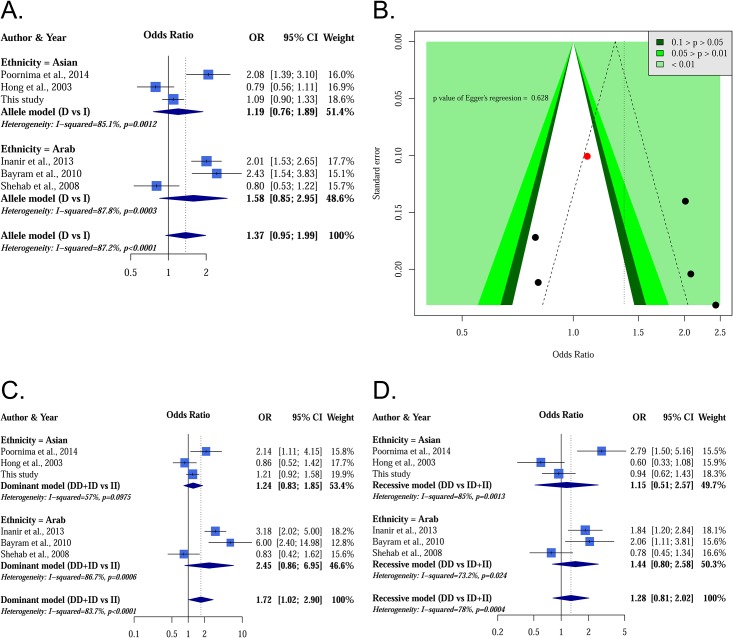
Selected results from the meta-analysis of angiotensin-converting enzyme insertion/deletion (ACE I/D) and knee osteoarthritis (OA). The top left subplot is a forest plot based on an allele model assumption (reference: I allele), and the top right subplot is a funnel plot based on the allele model assumption. The allele model is the most common method for detecting gene–disease associations; however, we found no significant signal in the allele model. However, the funnel plot indicates good symmetry in this meta-analysis. Results obtained with the dominant and recessive models are presented at the bottom. All results were nonsignificant.

Fig 2 presents selected results from our meta-analysis. None of the ACE alleles were associated with a significantly increased knee OA risk (p value = 0.092), and this result remained consistent for both subgroups (p values for Asians and Arabs: 0.445 and 0.148, respectively). Funnel plots were used to demonstrate the association between OR and standard error in the allele model, with each point representing a study. We found no evidence of asymmetry from a visual observation, and Egger’s regression yielded the same result (p value of Egger’s regression: 0.628). Other selected results were based on dominant and recessive models. In all classical genetic model analysis, the risk of knee OA for individuals carrying different ACE I/D genotype was not significantly higher than other genotypes in either the entire meta-analysis or the subgroup analyses. Other symmetrical assessments of the genotype, including dominant and recessive models, are shown in S1 Fig. Egger’s regression test indicated no evidence of publication bias among the included studies and all genetic models in this meta-analysis. A summary of the all results is shown in Table 4. Post hoc analysis shows a negative finding in all comparisons after multiple comparison correction. Moreover, all I^2^ values of classical genetic models exceeded 57%. This indicated a high heterogeneity on the association between ACE I/D on knee OA.

**Table 4:** Odds ratios of angiotensin-converting enzyme insertion/deletion (I/D) and knee osteoarthritis using assumptions from allele type, genotype, dominant, and recessive models.

Model	Total				Asian				Arab			
	OR (95% CI)	p value	I^2^	Egger's test	OR (95% CI)	p value	I^2^	Egger's test	OR (95% CI)	p value	I^2^	Egger’s test
Classical model^a^												
Allele (D vs I)	1.37(0.95–1.99)	0.092	87.2%	0.628	1.19(0.76–1.89)	0.445	85.1%	0.777	1.58(0.85–2.95)	0.148	85.1%	0.820
Dominant (DD + ID vs II)	1.72(1.02–2.90)	0.042	83.7%	0.440	1.24(0.83–1.85)	0.295	57.0%	0.815	2.45(0.86–6.95)	0.093	57.0%	0.978
Recessive (DD vs II + ID)	1.28(0.81–2.02)	0.289	78.0%	0.925	1.15(0.51–2.57)	0.735	85.0%	0.819	1.44(0.80–2.58)	0.228	73.2%	0.842
Post hoc analysis^b^												
Genotype (ID vs II)	1.65(1.06–2.56)	0.025	71.5%	0.500	1.22(0.96–1.54)	0.101	0.0%	0.976	2.46(1.10–5.49)	0.028	0.0%	0.922
Genotype (DD vs ID)	1.06 (0.74–1.53)	0.738	56.8%	0.795	1.05(0.50–2.20)	0.895	79.6%	0.755	1.12 (0.80–1.58)	0.499	0.0%	0.687
Genotype (DD vs II)	1.81(0.88–3.70)	0.106	86.6%	0.543	1.28(0.53–3.07)	0.585	83.3%	0.756	2.60(0.79–8.55)	0.115	83.3%	0.889

I^2^: index for assessing heterogeneity; value >50% indicates a moderate to high heterogeneity.

Egger's test: p value of Egger's regression for asymmetry assessment.

^a^: The significance level in the classical model was set as 0.05;

^b^: The significant level in post hoc analysis was set as 0.017 (corrected by the Bonferroni method).

Table 5 shows the results of a meta-regression that explored the source of the high heterogeneity. All of the potential factors could not significantly explain heterogeneity in the meta-analyses of all classical genetic models and post hoc analysis.

**Table 5:** Meta-regression analysis of heterogeneity.

Moderators	OR_interaction_ (95% CI)	p-value	τ^2^	R^2^
Classical model^c^				
Allele (D vs I)			0.1801^a^/0.1686^b^ (null model)	
Ethnicity (Asian is reference)	1.32(0.62–2.80)	0.466	0.1870^a^	0.0%
Gender (Female is reference)	1.50(0.02–125.15)	0.857	0.2541^a^	0.0%
Mean age (per 10 years)	0.80(0.53–1.20)	0.283	0.2032^a^	0.0%
Mean BMI (per 5 kg/m^2^)	1.23(0.59–2.57)	0.575	0.2219	0.0%
Dominant (DD + ID vs II)			0.3372^a^/0.2468^b^ (null model)	
Ethnicity (Asian is reference)	1.88(0.71–5.02)	0.207	0.2859^a^	15.2%
Gender (Female is reference)	1.10(0.00–572.15)	0.974	0.4905^a^	0.0%
Mean age (per 10 years)	0.78(0.43–1.44)	0.434	0.4516^a^	0.0%
Mean BMI (per 5 kg/m^2^)	1.18(0.45–3.12)	0.732	0.3660^b^	0.0%
Recessive (DD vs II + ID)			0.2519^a^/0.2900^b^ (null model)	
Ethnicity (Asian is reference)	1.25(0.46–3.38)	0.656	0.3089^a^	0.0%
Gender (Female is reference)	1.22(0.01–253.13)	0.942	0.3487^a^	0.0%
Mean age (per 10 years)	0.72(0.46–1.12)	0.140	0.2120^a^	15.8%
Mean BMI (per 5 kg/m^2^)	1.54(0.60–3.96)	0.374	0.3467^b^	0.0%
Post hoc analysis^d^				
Genotype (ID vs II)			0.1970^a^/0.1037^b^ (null model)	
Ethnicity (Asian is reference)	2.05(1.06–4.01)	0.034	0.0728^a^	63.0%
Gender (Female is reference)	0.49(0.00–79.34)	0.782	0.2631^a^	0.0%
Mean age (per 10 years)	0.88(0.53–1.46)	0.629	0.2888^a^	0.0%
Mean BMI (per 5 kg/m^2^)	1.10(0.51–2.36)	0.808	0.1732^b^	0.0%
Genotype (DD vs ID)			0.1148^a^/0.1699^b^ (null model)	
Ethnicity (Asian is reference)	1.05(0.47–2.38)	0.900	0.1667^a^	0.0%
Gender (Female is reference)	0.99(0.01–70.31)	0.998	0.1695^a^	0.0%
Mean age (per 10 years)	0.75(0.55–1.03)	0.078	0.0630^a^	45.1%
Mean BMI (per 5 kg/m^2^)	1.56(0.77–3.19)	0.219	0.1435^b^	15.5%
Genotype (DD vs II)			0.6795^a^/0.6342^b^ (null model)	
Ethnicity (Asian is reference)	2.01(0.47–8.57)	0.345	0.6975^a^	0.0%
Gender (Female is reference)	0.94(0.00–4356.45)	0.989	0.9402^a^	0.0%
Mean age (per 10 years)	0.65(0.30–1.39)	0.263	0.7236^a^	0.0%
Mean BMI (per 5 kg/m^2^)	1.59(0.38–6.58)	0.522	0.8404^b^	0.0%

^a^: These results were calculated from six studies;

^b^: These results were calculated from five studies because one study did not provide body mass index (BMI) information;

^c^: The significant level in the classical model was set as 0.05;

^d^: The significant level in post hoc analysis was set as 0.017 (corrected by the Bonferroni method).

OR_interaction_: interaction effect calculated by meta-regression; positive direction indicates that possible moderators might strengthen the knee OA risk in genetic variants relative to wild type. τ^2^: random effect variance in each model. R^2^: proportion of heterogeneity explainable by a specific moderator; this can be calculated using the following equation (negative value will be replaced by 0):
$${\text{R}}^{\text{2}}=\frac{\tau^{2}(null)-\tau^{2}(new)}{\tau^{2}(null)}$$

## 4. Discussion

Our case-control study results showed no significant associations between ACE I/D and knee OA. A meta-analysis of the associations in six studies yielded the same results. In our hypothesis, the ACE I/D DD genotype was associated with higher gene expression [63] and serum ACE levels [64] than the ID genotype, which was then followed by the II genotype. High blood ACE levels might increase bradykinin metabolism [26–29], thereby reducing the risk of knee OA because bradykinin involves the generation of pain, swelling, and cellular damage associated with joint disease [19–22]. According to this mechanism, the D allele may therefore be a protective factor against knee OA, although this was not confirmed in our study. In short, we considered our analysis provided a negative finding.

The main function of ACE is to convert angiotensin I to the potent vasoconstrictor angiotensin II [24,25], and we considered that this pathway might also impact susceptibility to knee OA. Knee OA is also considered an inflammation-related disease [9], and previous studies have confirmed a relationship between inflammation-related gene polymorphisms and knee OA [34,35,65,66]. Angiotensin II regulates the synthesis of proinflammatory cytokines, including TNF-alpha, IL-6, MCP-1, and NF-kappaB, and these proinflammatory cytokines play a key role in OA progression [67]. According to this mechanism, a higher ACE concentration might also increase the risk of knee OA. ACE expression is upregulated in the synovial stroma in rheumatoid arthritis, thereby contributing to synovial hypoxia and proliferation [68,69]; rheumatoid arthritis patients have higher ACE concentrations when compared with healthy controls [70]. As observed in patients with rheumatoid arthritis, patients with OA have higher levels of ACE activity in synovial fluid than controls [71]. The above evidence expounds upon the relationship between ACE and knee OA and includes at least two molecular pathways with opposite effects. Therefore, the effect of the ACE I/D D allele on knee OA might exert two opposing forces, and many possible factors could modify its effect. According to this complex mechanism, we considered the high heterogeneity in our meta-analysis to be reasonable, although further research is needed.

**Fig 3:**
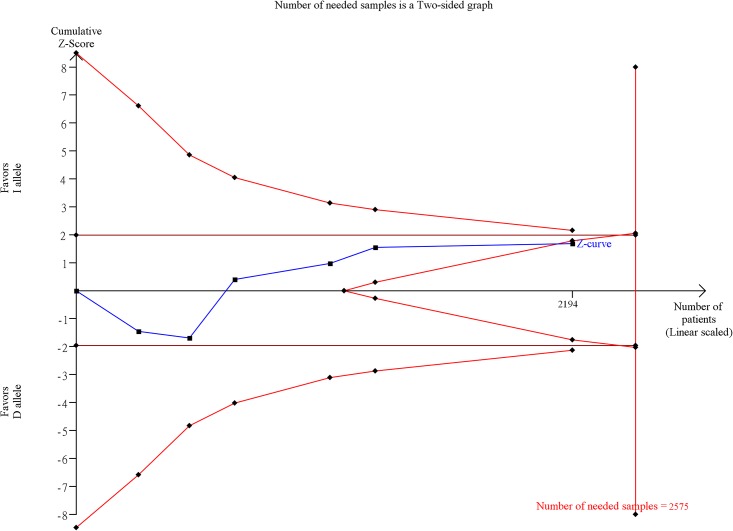
Trial Sequential Analysis (TSA) in this meta-analysis. TSA is a methodology that includes a sample size calculation for a meta-analysis with the threshold of statistical significance. We performed a TAS using an allele model assumption, but replaced the allele count with the sample size (divided by 2). Detailed settings: Significance level = 0.05; Power = 0.95; ratio of controls to cases = 1; hypothetical proportion of controls with D allele = 49; least extreme OR to be detected = 1.5; I^2^ (heterogeneity) = 90%.

We performed a Trial Sequential Analysis (TSA) for calculating the required sample size in this issue [72]. The settings used were as follows: a two-sided test with a power = 0.95 (because our result is negative, so we used a higher power to avoid false negatives) at a significance level of 0.05, ratio of controls to cases = 1, hypothetical proportion of controls with exposure = 49 (based on data), the lowest extreme OR to be detected = 1.5, and I^2^ (heterogeneity) = 90%. Following these settings, the number of needed samples is equal to 2575 and we collected 2194 samples in this meta-analysis. Although this was still insufficient, Fig 3 shows the last point is in the invalid area. Thus, we considered this meta-analysis provided negative evidence of ACE I/D on knee OA. High heterogeneity in the meta-analysis suggests that complex molecular pathways in ACE I/D and knee OA likely exist. Epistasis analysis is therefore necessary for explaining this heterogeneity.

Despite the high heterogeneity in the current study, our case–control study results were most similar to those reported by Hong et al. [36]. We considered this to be due to the high genetic homogeneity. Korea and Taiwan are both East Asian countries and share a similar ancestral population. We used the Cochrane Q test to compare these two studies in all genetic models and only obtained nonsignificant results. However, the Asian subgroup also exhibited high heterogeneity in the meta-analysis. This heterogeneity might have been due to a study of an Indian population [40]. The 1,000-Genome Project considered South Asians and East Asians to comprise different racial categories [42]. Because the relationship between ACE I/D and knee OA involves a complex mechanism, a small racial difference might also increase heterogeneity. We think that additional future studies conducted in neighboring regions will help us to control the population stratification.

Compared with the other included populations, our study population had the highest mean age, highest proportion of men, and lowest mean BMI. However, we considered that these differences in population characteristics might not affect the association between ACE I/D and knee OA because the results of a meta-regression showed that age, gender, and BMI could not explain the heterogeneity. Therefore, a high heterogeneity, regardless of the inclusion of Asian studies or Arab studies, might be due to another unknown factor. Because the role of ACE in knee OA may involve many possible pathways that even include opposing effects, we considered that the source of heterogeneity might include molecular factors such as gene–gene interactions. In fact, complex gene–gene interactions in the RAS were reported in the context of other diseases [73], which involved gene polymorphisms of ACE I/D. Future studies should further investigate the epistasis analysis of ACE I/D and knee OA [74].

In sensitivity analysis, we found none of the Arab population studies passed the HWE test. Previous studies reported that genotyping error was the most common reason for this HWE failure [75–77]. Although we could not confirm this reason, it suggests a lower evidence level in the Arab population studies. A meta-analysis remaining Asian studies yielded a nonsignificant result [OR: 1.22 (95% CI: 0.96–1.54, p value: 0.101)], this is a type of sensitivity analysis conducted by removing studies without HWE [57,58]. In summary, the sensitivity analysis shows the same results compared with original result.

Although we investigated the most common polymorphism in ACE, a previous study suggested that this might not be a functional locus. The functional polymorphism is most likely located between intron 18 and the 3′ UTR [78], and other commonly loci or unobserved ACE variants may capture the effects of functional variants on such local ACE actions more effectively. However, a very few genetic association studies have been carried out to investigate the association between these loci and OA, so we were unable to collect enough information in our meta-analysis. In addition to epistasis analysis, future efforts should be made for collecting a large sample to allow testing of more candidate genes, including the structural variation, to find additional putative functional variants, or an analysis at the genome-wide level.

Several potential limitations should be acknowledged. First, we relied on the tabular data for the meta-analysis, rather than on the individual patient data. However, we included a case–control study to improve the sample size and evidence level. Second, in addition to our case–control study, only five studies had investigated the link between OA and the ACE I/D polymorphism. The relationship between ACE I/D and knee OA involves a complex mechanism, thus indicating the need for additional studies. Our case–control study involves the largest sample to date and thus provides more evidence for a clear understanding of this association.

In conclusion, our meta-analysis provides evidence important to an exploration of the association between ACE I/D and knee OA. The ACE I/D D allele might not be protective against knee OA, and negative results revealed ACE I/D was not directly associated with OA. The high level of heterogeneity suggests a complex mechanism, and several possible environmental factors, including gender, age, and BMI, cannot explain this heterogeneity. This unexplainable heterogeneity might be related to gene–gene interactions although additional well-designed studies to address the relationship between ACE I/D and knee OA are needed to explore the underlying mechanism. An epistasis analysis of ACE I/D and knee OA should be conducted. A potential gene–environmental interaction should not be excluded, and additional environmental factors should also be investigated.

## Supporting Information

S1 Fig Funnel plots of the genotype, dominant, and recessive models. (PDF)

S1 Table PRISMA 2009 Checklist. (DOC)

S2 Table Meta-analysis on Genetic Association Studies Checklist | PLOS ONE. (DOCX)

S3 Table Search strategies and detailed records. (DOCX)

S4 Table Angiotensin-converting enzyme insertion/deletion (I/D) genotype frequencies in advanced OA, early OA, and controls. (DOC)
