# Supplementary material for: Angiotensin-Converting Enzyme Insertion/Deletion Polymorphism and Susceptibility to Osteoarthritis of the Knee: A Case-Control Study and Meta-Analysis
Source: PLoS One. 2016 Sep 22;11(9):e0161754. doi: 10.1371/journal.pone.0161754 (PMC5033346; doi:10.1371/journal.pone.0161754)
Supplement: S1 Fig — (PDF) [file pone.0161754.s001.pdf]

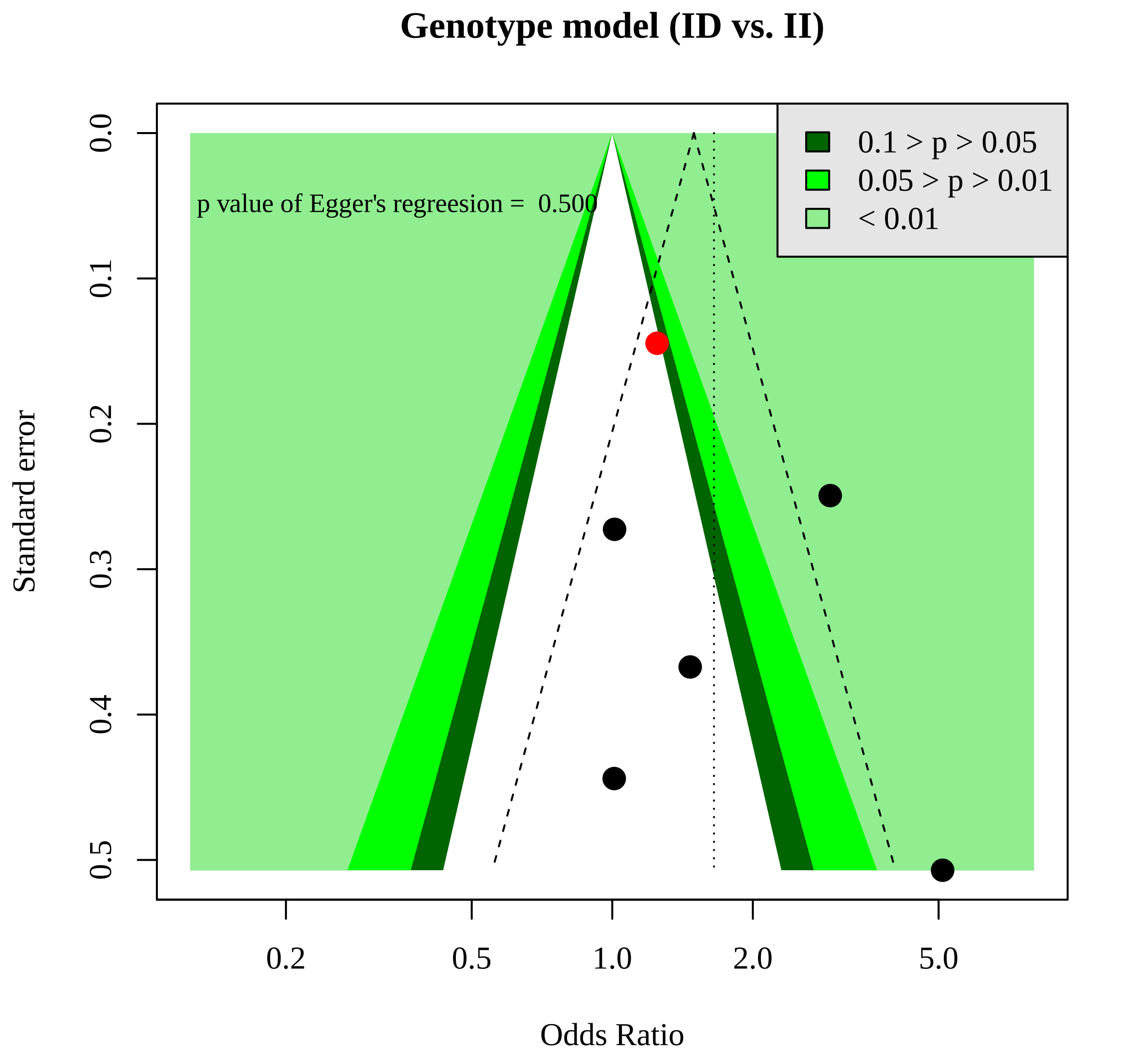
Genotype model (ID vs. II)
p value of Egger's regreesion = 0.500
0.1 > p > 0.05
0.05 > p > 0.01
< 0.01
Standard error
0.0
0.1
0.2
0.3
0.4
0.5
0.2
0.5
1.0
2.0
5.0
Odds Ratio

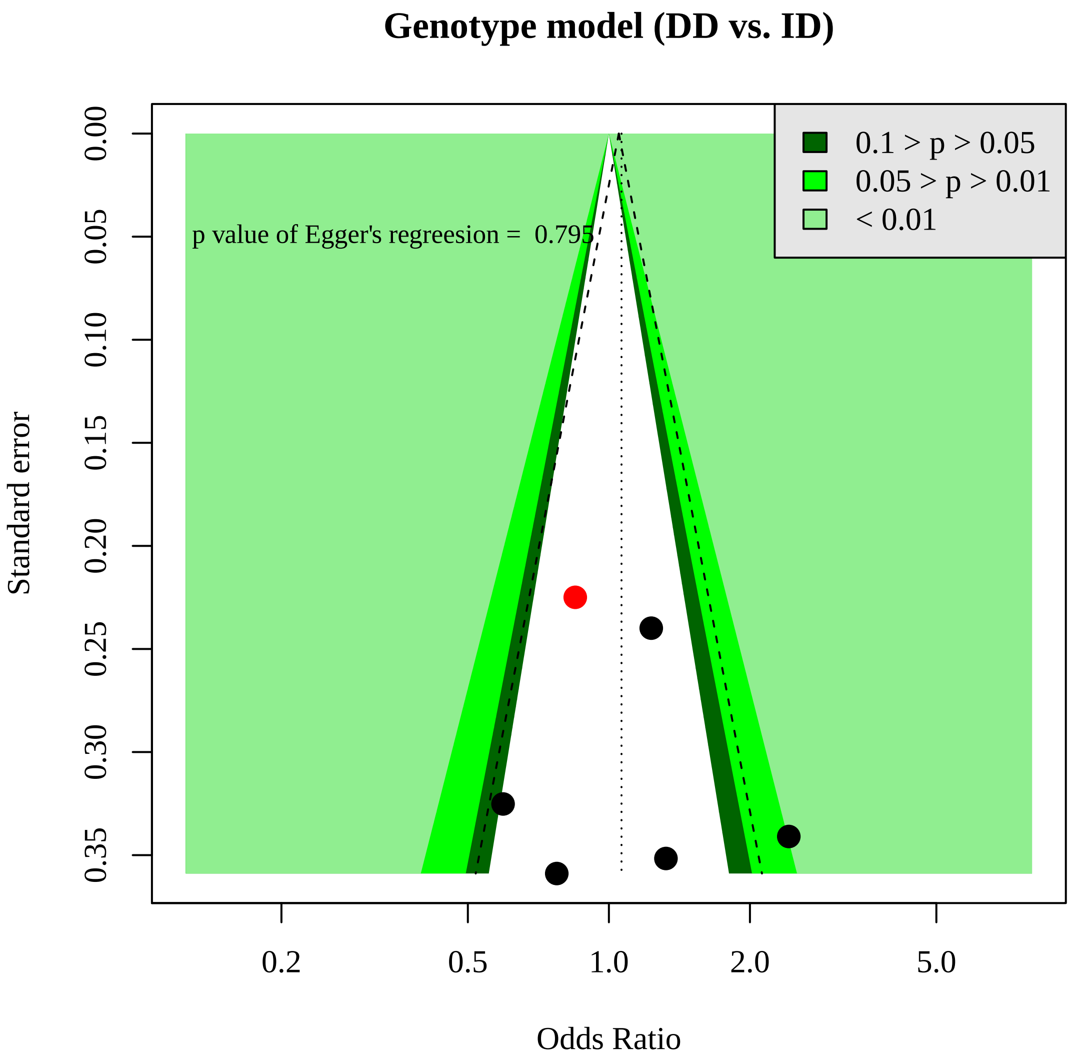
Genotype model (DD vs. ID)
0.1 > p > 0.05
0.05 > p > 0.01
< 0.01
p value of Egger's regreesion = 0.795
Standard error
0.00
0.05
0.10
0.15
0.20
0.25
0.30
0.35
0.2
0.5
1.0
2.0
5.0
Odds Ratio

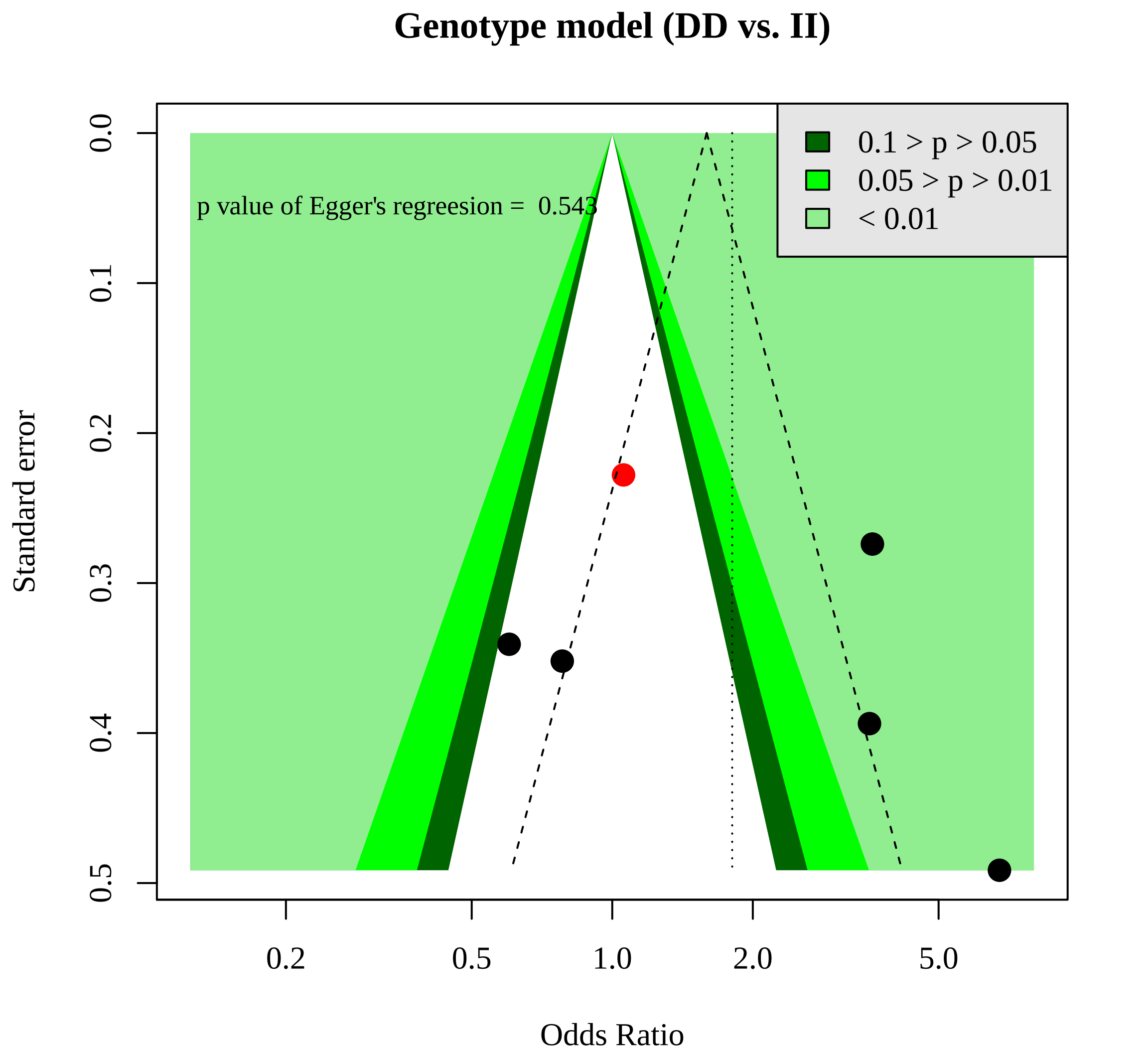
Genotype model (DD vs. II)
0.1 > p > 0.05
0.05 > p > 0.01
< 0.01
p value of Egger's regreesion = 0.543
Standard error
0.0
0.1
0.2
0.3
0.4
0.5
0.2
0.5
1.0
2.0
5.0
Odds Ratio

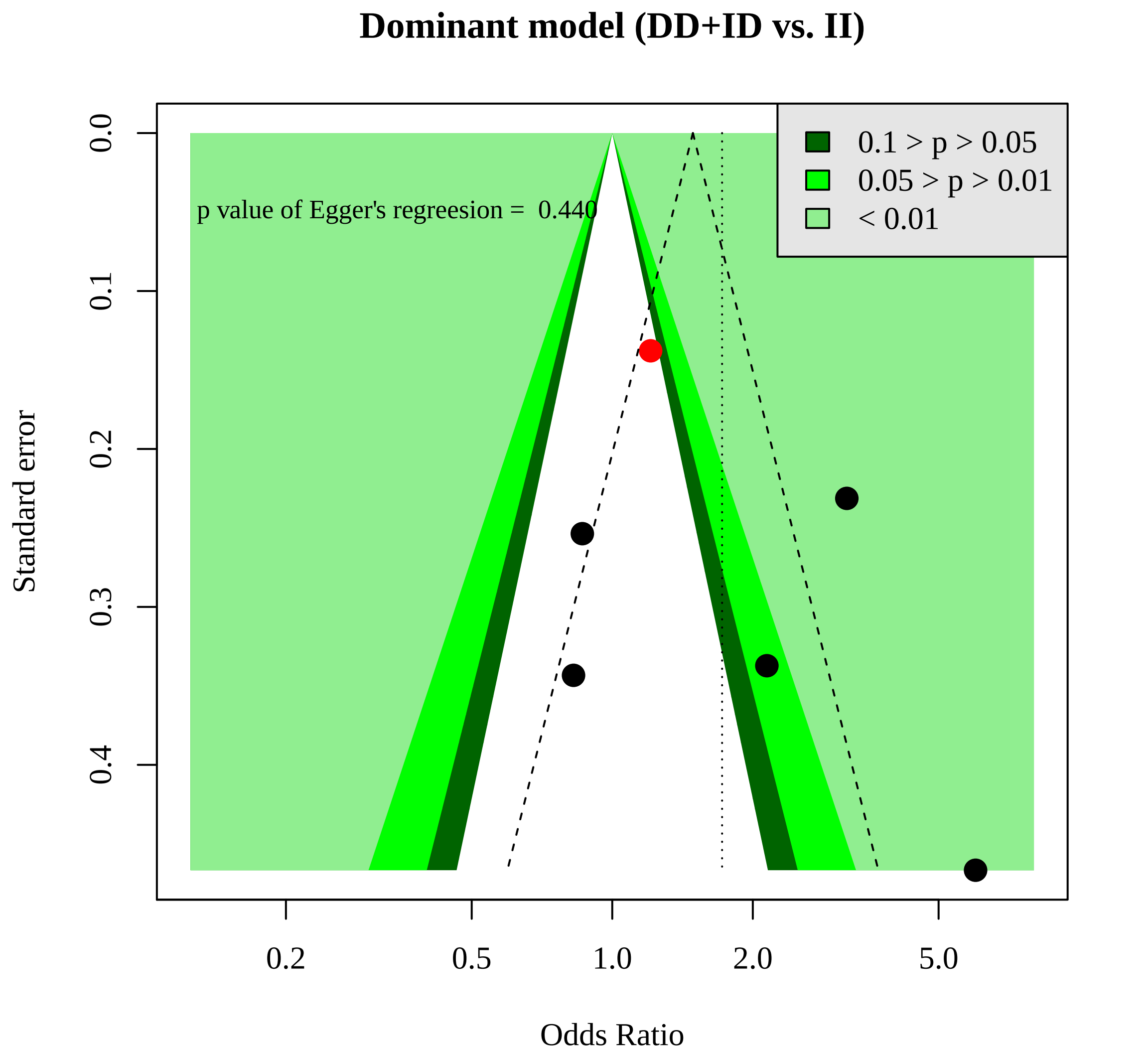
Dominant model (DD+ID vs. II)
p value of Egger's regreesion = 0.440
0.1 > p > 0.05
0.05 > p > 0.01
< 0.01
Standard error
0.0
0.1
0.2
0.3
0.4
Odds Ratio
0.2
0.5
1.0
2.0
5.0

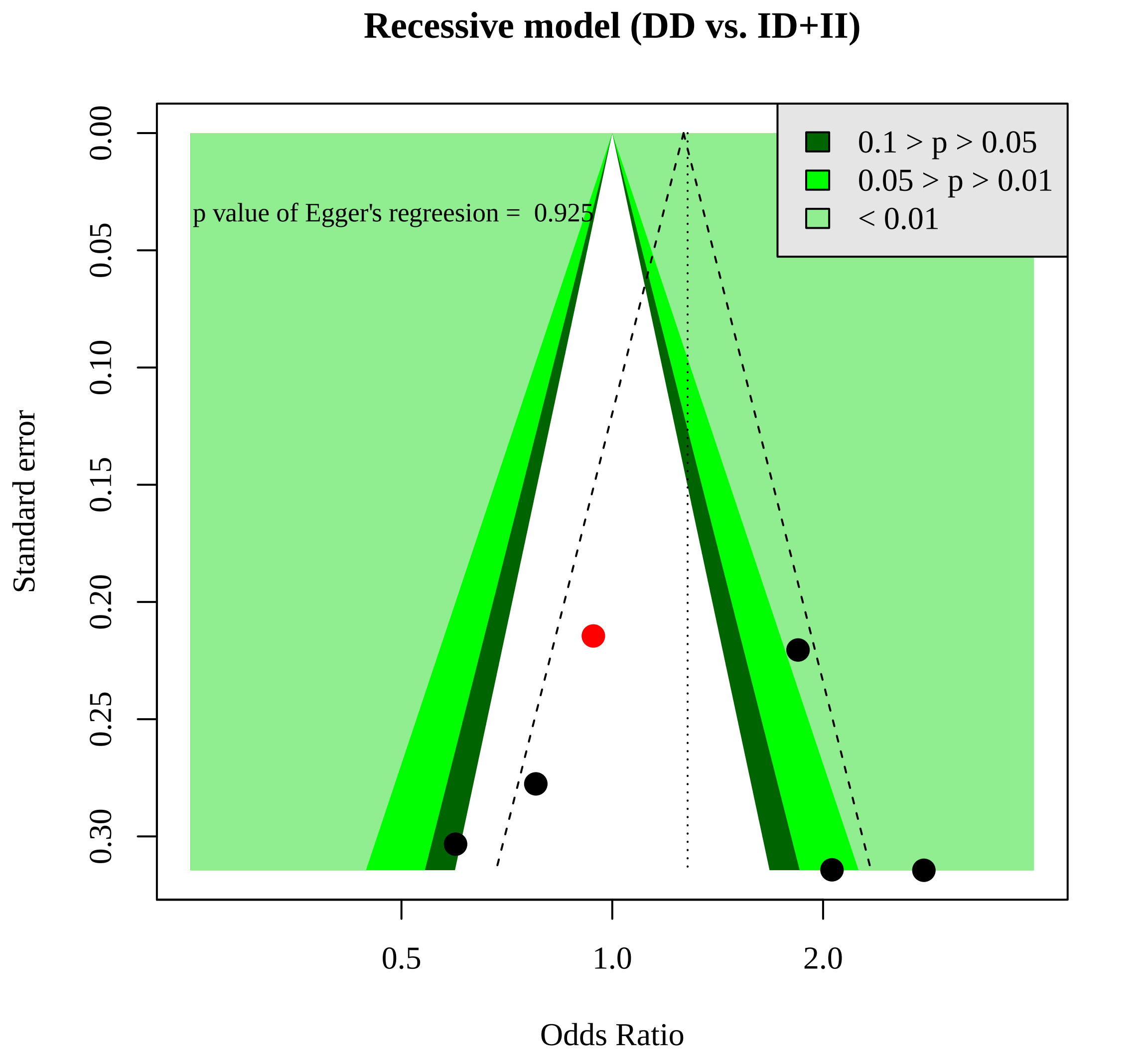

Recessive model (DD vs. ID+II)
0.1 > p > 0.05
0.05 > p > 0.01
< 0.01
p value of Egger's regreesion = 0.925
Standard error
0.00
0.05
0.10
0.15
0.20
0.25
0.30
0.5
1.0
2.0
Odds Ratio
