# Supplementary material for: Angiotensin-Converting Enzyme Insertion/Deletion Polymorphism and Susceptibility to Osteoarthritis of the Knee: A Case-Control Study and Meta-Analysis
Source: PLoS One. 2016 Sep 22;11(9):e0161754. doi: 10.1371/journal.pone.0161754 (PMC5033346; doi:10.1371/journal.pone.0161754)
Supplement: S3 Table — (DOCX) [file pone.0161754.s004.docx]

**S2 Table. Search strategies and detailed records.**

| **Relevant text of ACE I/D**   1. Peptidyl-Dipeptidase A 2. Angiotensin Converting Enzyme 3. Angiotensin I-Converting Enzyme 4. Antigens, CD143 5. Carboxycathepsin 6. CD143 Antigens 7. Dipeptidyl Peptidase A 8. Kininase A 9. Kininase II 10. ACE 11. I/D 12. insertion/deletion 13. rs4340 14. rs1799752 15. rs13447447 16. rs4646994 17. ((1 or 2 or 3 or 4 or 5 or 6 or 7 or 8 or 9 or 10) and (11 or 12)) or 13 or 14 or 15 or 16 | **Relevant text of knee osteoarthritis**   1. Arthritis, Degenerative 2. Osteoarthrosis 3. Osteoarthrosis Deformans 4. osteoarthritis 5. OA 6. 18 or 19 or 20 or 21 or 22   **Combined (Final strategy)**   1. 17 and 23 |
| --- | --- |

Web sites and uniform resource locator:

**PUBMED**: <http://www.ncbi.nlm.nih.gov/pubmed>

**EMBASE**: https://www.embase.com

**Records from PUBMED [1-17]:**

*Unrelated records (wrong outcome [3-5,7,8,10-13,15-17])*

*Included studies [1,2,6,9,14]*

**Records from EMBASE [1,2,6,9,14,18-20] (1 of record is a duplicate record [1]):**

*Duplicated records [1,2,6,9,14]*

*Unrelated records (wrong outcome [18,20])*

*Paper didn’t provide detailed genotyping data [19]*

**Reference**

1. Poornima S, Subramanyam K, Khan IA, Hasan Q. The insertion and deletion (I28005D) polymorphism of the angiotensin I converting enzyme gene is a risk factor for osteoarthritis in an Asian Indian population. Journal of the renin-angiotensin-aldosterone system : JRAAS. 2015;16(4):1281-7. Epub 2014/09/03. doi: 10.1177/1470320314547403.

2. Inanir A, Yigit S, Tural S, Cecen O, Yildirim E. MTHFR gene C677T mutation and ACE gene I/D polymorphism in Turkish patients with osteoarthritis. Disease markers. 2013;34(1):17-22. Epub 2012/10/24. doi: 10.3233/dma-2012-00939.

3. Ahmed AZ, El-Shahaly HA, Omar AS, Ghattas MH. Patterns of angiotensin converting enzyme insertion/deletion gene polymorphism among an Egyptian cohort of patients with rheumatoid arthritis. International journal of rheumatic diseases. 2013;16(3):284-90. Epub 2013/08/29. doi: 10.1111/j.1756-185X.2012.01820.x.

4. Yigit S, Inanir A, Tural S, Ates O. Association of angiotensin converting enzyme (ACE) gene I/D polymorphism and rheumatoid arthritis. Gene. 2012;511(1):106-8. Epub 2012/09/25. doi: 10.1016/j.gene.2012.09.036.

5. Inanir A, Yigit S, Tural S, Ozturk SD, Akkanet S, Habiboglu A. Significant association between insertion/deletion polymorphism of the angiotensin-convertig enzyme gene and ankylosing spondylitis. Molecular vision. 2012;18:2107-13. Epub 2012/08/10.

6. Bayram B, Sayin E, Gunes HV, Degirmenci I, Turkoglu Z, Doganer F, et al. DD genotype of ace gene I/D polymorphism is associated in a Turkish study population with osteoarthritis. Molecular biology reports. 2011;38(3):1713-6. Epub 2010/09/17. doi: 10.1007/s11033-010-0284-y.

7. Coto-Segura P, Alvarez V, Soto-Sanchez J, Morales B, Coto E, Santos-Juanes J. Lack of association between angiotensin I-converting enzyme insertion/deletion polymorphism and psoriasis or psoriatic arthritis in Spain. International journal of dermatology. 2009;48(12):1320-3. Epub 2009/11/26. doi: 10.1111/j.1365-4632.2009.04245.x.

8. Shehab DK, Al-Jarallah KF, Al-Awadhi AM, Al-Herz A, Nahar I, Haider MZ. Association of angiotensin-converting enzyme (ACE) gene insertion-deletion polymorphism with spondylarthropathies. Journal of biomedical science. 2008;15(1):61-7. Epub 2007/08/24. doi: 10.1007/s11373-007-9203-1.

9. Shehab DK, Al-Jarallah KF, Alawadhi AM, Al-Herz A, Nahar I, Haider MZ. Prevalence of angiotensin-converting enzyme gene insertion-deletion polymorphism in patients with primary knee osteoarthritis. Clinical and experimental rheumatology. 2008;26(2):305-10. Epub 2008/06/21.

10. Uppal SS, Haider MZ, Hayat SJ, Abraham M, Sukumaran J, Dhaunsi GS. Significant association of insertion/deletion polymorphism of the angiotensin-converting enzyme gene with rheumatoid arthritis. The Journal of rheumatology. 2007;34(12):2395-9. Epub 2007/11/07.

11. Saibeni S, Spina L, Virgilio T, Folcioni A, Borsi G, de Franchis R, et al. Angiotensin-converting enzyme insertion/deletion gene polymorphism in inflammatory bowel diseases. European journal of gastroenterology & hepatology. 2007;19(11):976-81. Epub 2007/12/01. doi: 10.1097/MEG.0b013e3282efa3fc.

12. Al-Awadhi AM, Hasan EA, Sharma PN, Haider MZ, Al-Saeid K. Angiotensin-converting enzyme gene polymorphism in patients with psoriatic arthritis. Rheumatology international. 2007;27(12):1119-23. Epub 2007/04/19. doi: 10.1007/s00296-007-0349-y.

13. Chou HT, Tsai CH, Tsai FJ. Association between angiotensin I-converting enzyme gene insertion/deletion polymorphism and risk of rheumatic heart disease. Japanese heart journal. 2004;45(6):949-57. Epub 2005/01/19.

14. Hong SJ, Yang HI, Yoo MC, In CS, Yim SV, Jin SY, et al. Angiotensin converting enzyme gene polymorphism in Korean patients with primary knee osteoarthritis. Experimental & molecular medicine. 2003;35(3):189-95. Epub 2003/07/15. doi: 10.1038/emm.2003.26.

15. Alsaeid K, Haider MZ, Ayoub EM. Angiotensin converting enzyme gene insertion-deletion polymorphism is associated with juvenile rheumatoid arthritis. The Journal of rheumatology. 2003;30(12):2705-9. Epub 2004/01/14.

16. Zapico I, Coto E, Rodriguez A, Alvarez C, Torre JC, Alvarez V. A DNA polymorphism at the alpha2-macroglobulin gene is associated with the severity of rheumatoid arthritis. The Journal of rheumatology. 2000;27(10):2308-11. Epub 2000/10/19.

17. Arnett DK, Borecki IB, Ludwig EH, Pankow JS, Myers R, Evans G, et al. Angiotensinogen and angiotensin converting enzyme genotypes and carotid atherosclerosis: the atherosclerosis risk in communities and the NHLBI family heart studies. Atherosclerosis. 1998;138(1):111-6. Epub 1998/07/25.

18. Mandal RK, Yaday SS, Panda AK, Khattri S. Insertion/deletion polymorphism of the ACE gene increased risk of Behcet disease: evidence from a meta-analysis. Annals of Saudi medicine. 2013;33(5):437-42. Epub 2013/11/06. doi: 10.5144/0256-4947.2013.437.

19. SAYIN BE, BAYRAM B, TÜRKOĞLU Z, KELEŞTEMUR Ü, MUTLU F. Angiotensin converting enzyme (ACE) gene I/D polymorphism genotypes and ACE levels of serum and synovial fluid of patients with osteoarthritis. FABAD J Pharm Sci. 2009;34:77-81.

20. Zhang J, Sun X, Weng Q, Lv S. Relationship between angiotensin converting enzyme gene polymorphism and risk factors in cerebral infarction. Chin J Clin Neurosci. 2002;10(04):361-4.
