# Supplementary material for: Angiotensin-Converting Enzyme Insertion/Deletion Polymorphism and Susceptibility to Osteoarthritis of the Knee: A Case-Control Study and Meta-Analysis
Source: PLoS One. 2016 Sep 22;11(9):e0161754. doi: 10.1371/journal.pone.0161754 (PMC5033346; doi:10.1371/journal.pone.0161754)
Supplement: S4 Table — (DOC) [file pone.0161754.s005.doc]

**S4 Table. Angiotensin-converting enzyme insertion/deletion (I/D) genotype frequencies in advanced OA, early OA** and controls

|  | **Advanced OA** | **Early OA** | **Control** | **Odds Ratio^a^ (95% CI)** | **p value** | **Odds Ratio^b^ (95% CI)** | **p value** |
| --- | --- | --- | --- | --- | --- | --- | --- |
| **Allele** |  |  |  |  |  |  |  |
| I Allele | 115(63.9%) | 458(64.1%) | 559(66.1%) | 1 | 0.425 | 1 | 0.575 |
| D Allele | 65(36.1%) | 256(35.9%) | 287(33.9%) | 1.09(0.88-1.34) |  | 1.10(0.75-1.54) |  |
| **Genotype** |  |  |  |  | 0.419 |  | 0.429 |
| II | 34(37.8%) | 141(39.5%) | 185(43.7%) | 1 |  | 1 |  |
| ID | 47(52.2%) | 176(49.3%) | 189(44.7%) | 1.22 (0.90-1.65) |  | 1.35 (0.83-2.20) |  |
| DD | 9(10.0%) | 40(11.2%) | 49(11.6%) | 1.07 (0.67-1.72) |  | 1.00 (0.45-2.22) |  |
| **Dominant** |  |  |  |  | 0.232 |  | 0.300 |
| II | 34(37.8%) | 141(39.5%) | 185(43.7%) | 1 |  | 1 |  |
| DD + ID | 56(62.2%) | 216(61.5%) | 238(56.3%) | 1.19 (0.89-1.59) |  | 1.28 (0.80-2.04) |  |
| **Recessive** |  |  |  |  | 0.868 |  | 0.667 |
| II + ID | 81(90.0%) | 317(88.8%) | 374(88.4%) | 1 |  | 1 |  |
| DD | 9(10.0%) | 40(11.2%) | 49(11.6%) | 0.96 (0.62-1.50) |  | 0.85 (0.40-1.80) |  |

OA: osteoarthritis; Advanced OA: KL > 2; Early OA: KL = 2; Control: KL < 2.

^a^: Early OA vs. Control; ^b^: Advanced OA vs. Control; CI: confidence interval

The p value of global analysis^a^: 0.4156 (MAX3) and 0.4774 (GMS)

The p value of global analysis^b^: 0.5107 (MAX3) and 0.6150 (GMS)
